# Genome‐Wide Association Analyses of HPV16 and HPV18 Seropositivity Identify Susceptibility Loci for Cervical Cancer

**DOI:** 10.1002/jmv.70195

**Published:** 2025-01-31

**Authors:** Theresa Beckhaus, Linda Kachuri, Taishi Nakase, Peter Schürmann, Rieke Eisenblätter, Maya Geerts, Gerd Böhmer, Hans‐Georg Strauß, Christine Hirchenhain, Monika Schmidmayr, Florian Müller, Peter A. Fasching, Norman Häfner, Alexander Luyten, Matthias Jentschke, Peter Hillemanns, Tracy A. O'Mara, Stephen S. Francis, John S. Witte, Thilo Dörk, Dhanya Ramachandran

**Affiliations:** ^1^ Gynaecology Research Unit, Department of Gynecology and Obstetrics Hannover Medical School Hannover Germany; ^2^ Department of Epidemiology and Population Health Stanford University School of Medicine Stanford California USA; ^3^ Stanford Cancer Institute Stanford School of Medicine Stanford California USA; ^4^ IZD Hannover Hannover Germany; ^5^ Department of Gynaecology Martin‐Luther University Halle‐Wittenberg Germany; ^6^ Department of Gynaecology Clinics Carl Gustav Carus, University of Dresden Dresden Germany; ^7^ Department of Gynaecology Technical University of Munich Munich Germany; ^8^ Martin‐Luther Hospital Charite University Berlin Germany; ^9^ Department of Gynecology and Obstetrics Friedrich Alexander University of Erlangen–Nuremberg (FAU) Erlangen Germany; ^10^ Department of Gynecology Jena University Hospital, Friedrich‐Schiller‐University Jena Germany; ^11^ Dysplasia Unit, Department of Gynecology and Obstetrics Mare Klinikum Kronshagen Germany; ^12^ Department of Gynecology Wolfsburg Hospital Wolfsburg Germany; ^13^ Cancer Research Program QIMR Berghofer Medical Research Institute Brisbane Queensland Australia; ^14^ Department of Neurological Surgery, Weill Institute for Neuroscience University of California San Francisco San Francisco California USA; ^15^ Department of Biomedical Data Science and Department of Genetics Stanford University School of Medicine Stanford California USA

**Keywords:** cervical carcinoma, eQTL, human papillomavirus, infection, SNP

## Abstract

Infection by high‐risk human papillomavirus is known to exacerbate cervical cancer development. The host immune response is crucial in disease regression. Large‐scale genetic association studies for cervical cancer have identified few susceptibility variants, mainly at the human leukocyte antigen locus on chromosome 6. We hypothesized that the host immune response modifies cervical cancer risk and performed three genome‐wide association analyses for HPV16, HPV18 and HPV16/18 seropositivity in 7814, 7924, and 7924 samples from the UK Biobank, followed by validation genotyping in the German Cervigen case‐control series of cervical cancer and dysplasia. In GWAS analyses, we identified two loci associated with HPV16 seropositivity (6p21.32 and 15q26.2), two loci associated with HPV18 seropositivity (5q31.2 and 14q24.3), and one locus for HPV16 and/or HPV18 seropositivity (at 6p21.32). MAGMA gene‐based analysis identified *HLA‐DQA1* and *HLA‐DQB1* as genome‐wide significant (GWS) genes. In validation genotyping, the genome‐wide significant lead variant at 6p21.32, rs9272293 associated with overall cervical disease (OR = 0.86, *p* = 0.004, 95% CI = 0.78–0.95, *n* = 3710) and HPV16 positive invasive cancer (OR = 0.73, *p* = 0.005, 95% CI = 0.59–0.91, *n* = 1431). This variant was found to be a robust eQTL for *HLA‐DRB1*, *HLA‐DQB1‐AS1*, *C4B*, *HLA‐DRB5*, *HLA‐DRB6*, *HLA‐DQB1*, and *HLA‐DPB1* in a series of cervical epithelial tissue samples. We additionally genotyped twenty‐four HPV seropositivity variants below the GWS threshold out of which eleven variants were found to be associated with cervical disease in our cohort, suggesting that further seropositivity variants may determine cervical disease outcome. Our study identifies novel genomic risk loci that associate with HPV type‐specific cervical cancer and dysplasia risk and provides evidence for candidate genes at one of the risk loci.

**Abbreviations**
ANOVA: analysis of variance
CI: confidence interval
CIN: cervical intraepithelial neoplasia
DICE: database of immune cell expression, expression quantitative trait loci and epigenomics
eQTL: expression quantitative trait locus
GWS: genome‐wide significance
HPV: human papillomavirus
HSIL: high‐grade squamous intraepithelial lesion
HWE: Hardy‐Weinberg equilibrium
LD: linkage disequilibrium
LSIL: low‐grade squamous intraepithelial lesion
OR: odds ratio

## 1 Introduction

Cervical cancer is the fourth most common cancer in women worldwide and approximately 2100 women in Germany currently die of cervical cancer every year [1, 2]. Persistent HPV infection [3], especially with high risk HPV types (including HPV16, HPV18, HPV31, HPV33, HPV35, HPV45, HPV52 and HPV58), is known to trigger cervical dysplasia and progression to cervical cancer, but not every woman infected with HPV develops invasive cervical cancer [4]. This suggests the involvement of environmental factors (such as the use of oral contraceptives, infection with *Chlamydia trachomatis* and smoking) [5] and genetic factors that may increase the risk for invasive cervical disease [6]. Genome wide association studies (GWASs) have previously identified multiple susceptibility loci for cervical cancer, mainly at the human leukocyte antigen (HLA) locus on chromosome 6 that is involved in the host immune response [7, 8, 9, 10, 11, 12]. Follow‐up studies indicated that genes from both the MHC I and II regions are modulated by cervical cancer risk variants [13]. Additional non‐*HLA* susceptibility loci have been identified on chromosome 2 (*PAX8*) [10], chromosome 5 (*TERT/CLPTM1L*) [11], and chromosome 17 (*GSDMB*) [8] that have been validated in further cohorts in meta‐analyses [6, 11, 12]. The hitherto known risk variants, however, do not explain all of the genetic heritability for this disease and further susceptibility loci remain to be discovered. Given the specific role of HPV in cervical cancer, GWASs for HPV seropositivity may inform HPV‐type associated cervical cancer risk [9, 14, 15]. Notably, two population‐derived GWASs have linked variants at chromosome 6 (*HLA*) and chromosome 14 (near *VASH1*) with HPV16 and HPV18 seropositivity, respectively, and these may also constitute cervical cancer risk variants [14, 16]. In the present study we aimed to validate variants arising from HPV16 and/or HPV18 seropositivity GWASs of the UK Biobank in our German Cervigen cohort of cervical cancers and dysplasias.

## 2 Methods

### 2.1 GWAS Analyses

GWAS summary statistics for HPV were generated in European ancestry UKB participants as previously described (Kachuri et al., 2020). GWAS analyses were conducted with approved access to UK Biobank data under application number 14105. Briefly, seropositivity was determined based on established thresholds [17] for each HPV individual antigen and this was used to derive the following phenotypes: HPV16 E6/E7/L1 positive (*n* = 674) versus no HPV detected (*n* = 7140), HPV18 L1 positive (*n* = 191) versus HPV18 negative (*n* = 7733), or HPV16/18+ (HPV16 E6/E7/L1 or HPV18 L1, *n* = 784) versus no HPV detected (*n* = 7140) (Figure S1, Table S1). The slightly different design for HPV18 was due to the observation of several HPV16/HPV18 double positives which led us to also leave HPV16 only carriers in the HPV18‐negative group. Logistic regression models were adjusted for age at sample collection, sex, serology assay date, top 10 genetic ancestry principal components, and genotyping array. Variants at *p* < 5 × 10E‐6 were submitted to LDlink (https://ldlink.nih.gov/) and independent SNPs at (*R* ^2^ < 0.3) were taken for PCR validation genotyping in the German Cervigen cohort as described below. Variants with a minor allele frequency (MAF) < 0.01 in 1000 G EUR in LDlink, as far as known at the time of analysis, were filtered out. We noticed *post hoc* that some variants had different MAFs in dbSNP (https://www.ncbi.nlm.nih.gov/snp/) but they were retained since they had been genotyped. In total, 170, 193, and 677 variants across the three analyses, respectively, were identified at *p* < 5 × 10E‐6. From this, 29 variants were taken for genotyping (Table S2).

### 2.2 Patient Material

In order to investigate whether variants arising from HPV seropositivity GWASs can influence the risk for cervical cancer or dysplasia, we performed a case‐control genotyping study on a total of 3710 samples from the German Cervigen consortium [18]. This included 2448 cases with either invasive cervical cancer or cervical dysplasia, acquired from nine German hospitals in Hannover, Wolfsburg, Jena, Erlangen, Dresden, Halle, Munich, Berlin and Bad Münder. The median age at diagnosis for cases overall was 41 years (range 17–78 years). Additionally, 1203 cancer‐ and dysplasia‐free healthy females (with unknown seropositivity status) at Hannover Medical School served as the control group. The median age for control samples was 37 years (range 18–89 years). We utilized all cancer/dysplasia‐free controls at hand (with unknown serostatus) since the primary aim of our study was to identify cervical cancer risk associated SNPs. After obtaining informed consent, 5 mL peripheral venous blood was drawn. The study was approved by the Ethics committee of Hannover Medical School (Votes No. 441 and 10737). All samples and data that have been used were in accordance with German medical council regulations.

A second patient‐derived cohort of 303 cervical smears from Hannover Medical School was used in eQTL analysis as explained in the section “Transcript analysis”. All these samples were cancer/dysplasia free. Genomic DNA was at hand from 280 of these cervical smears from which 78 samples were HPV positive and 202 were HPV negative. In terms of HPV positivity, 33 samples were infected by HPV16, 9 samples contained HPV18, and 36 samples had other strains of hrHPV (31, 33, 35, 39, 45, 51, 52, 56, 58, 59, 66, or 68) as determined with the RealTime High Risk HPV test on the Abbott m2000 PCR system. We note a high correlation between HPV positivity and the detection of cytological lesions: 184/202 HPV‐negative samples were lesion‐negative (91%), while 54/78 HPV‐positive samples were also lesion‐positive (69%).

### 2.3 SNP Genotyping

Genomic DNA was isolated from peripheral white blood cells via standard phenol chloroform extraction. For lead variant rs9272293, a TaqMan assay was used for genotyping (Assay ID: C__30510445_10) the entire Cervigen case‐control series, also including samples with unknown HPV status (cervical cancer or dysplasia cases = 2448, cancer‐free controls (with unknown serostatus) = 1203). For the other 28 candidate variants, specific target amplification (STA) was performed on the DNA samples using pooled SNPtype assays (Fluidigm) and the 2× Multiplex PCR Master Mix (Qiagen). Simultaneous genotyping was then carried out using Fluidigm genotyping arrays on the BioMark HD system (Fluidigm) (Table S2). Cluster plots were visualized for each of these SNPs after genotyping (Figure S2). The 28 sub‐GWS variants were only genotyped in a part of the Cervigen cohort where HPV status was known for cases (cervical cancer or dysplasia cases = 1357, cancer‐free controls (with unknown serostatus) = 1203). Three variants were excluded from further evaluation (rs151043538, rs35812074 and rs551344817) due to poor or monomorphic clustering of their genotypes on the resulting scatter plots (Figure S2, Table S3). The remaining 25 variants and lead variant rs9272293 were forwarded to further statistical analyses.

### 2.4 Statistical Analysis

Call rates and concordance rates were calculated and the variants were tested for Hardy‐Weinberg equilibrium (HWE) using goodness‐of‐fit chi‐square tests (Table S3). One variant, rs12867177 failed HWE and was excluded from further statistical testing. For the remaining 25 variants, *p* values, odds ratios (OR) and 95% confidence intervals (CI) were calculated via logistic regression using the STATA v17 software (StataCorp) with case‐control status as the outcome and the variant genotype as the predictor variable. We carried out stratified analyses based on disease severity and HPV status. The cases were further divided into three groups: LSIL/low‐grade dysplasias (CIN1 combined with CIN2 patients at age < 30 years [CIN2 < 30]), HSIL/high‐grade dysplasias (CIN2 cases at age ≥ 30 years (CIN2 ≥ 30) combined with CIN3 patients), and invasive carcinomas. The group of invasive cervical carcinomas was further stratified by histological type into squamous cell carcinomas and adenocarcinomas. The subgroups HSIL and invasive carcinoma were also combined for joint analysis. For stratified analyses based on HPV type, samples were grouped into HPV16 positive, HPV18 positive, HPV16 and/or 18 positives (including both single and double positives), and “HPV other” positives (which included 12 other HPV types, detected mainly with the Abbott real time high risk HPV assay: HPV 31, 33, 35, 39, 45, 51, 52, 56, 58, 59, 66, and 68). Each of these groups was compared against cancer‐ and dysplasia‐ free controls.

In regard of multiple testing for twenty‐five SNPs and sixteen comparisons, a Bonferroni corrected *p* value of *p* < 0.00013 would be considered statistically significant. However, as we followed up variants from GWASs (although of HPV seropositivity), we considered association with two‐sided *p* values below 0.05 and the same direction of effect as confirmatory evidence of association with cervical disease in a particular subgroup in this genetic association study.

### 2.5 Bioinformatic Analysis

GWAS summary statistics were uploaded to the FUMA webtool [19] for MAGMA [20] analysis. Variants within a window of 25kbp were mapped to genes in the vicinity. Since variants were mapped to 19,380 protein coding genes, the Bonferroni corrected significance threshold was set at *α* = 0.05/19380 = 2.58 × 10E‐6. Gene‐set enrichment analysis was run as part of MAGMA and GENE2FUNC functionality within the FUMA webtool. We additionally submitted the variants +/− 1Mbp of the lead GWS variant (*p* < 5 × 10E‐8) for fine‐mapping analysis via SuSiE [21] and Rsparsepro [22] to identify credible sets within this locus.

In silico annotation was performed using HaploReg v4.2 [23], RegulomeDB [24] and ForgeDB [25] for chromatin state, transcription factor binding, known eQTL effects, and changes in regulatory motifs. DNA sequences flanking 10 bp around the lead variants (major/minor allele) were submitted to TOMTOM [26] (MEME Suitev5.3.3, using the HOCOMOCOv11_full_HUMAN database) to identify allele‐specific transcription factor binding sites. In parallel, 25 bp flanks around the SNPs of interest were submitted to the PERFECTOS‐APE webtool (https://opera.autosome.org/perfectosape using the HOCOMOCO11 HUMAN database) to determine allele‐specific transcription factor binding sites.

The lead variants were also annotated for the closest genes (+/− 1Mbp) using UCSC Genome Browser GRCh37. Additionally, the GTEx database (v8, www.gtexportal.org) was queried for known eQTLs or sQTLs in whole blood (Supplementary Table S4A). Similarly, the eQTLGen consortium's eQTL database (https://eqtlgen.org/cis-eqtls.html) was queried for whole blood eQTLs as well (Table S4A and Table S5).

Since the variants arose from HPV seropositivity GWASs and may mediate the host immune response to HPV infection, we also explored possible eQTL effects in immune cells within the DICE database [27] (www.dice-database.org) and investigated the twelve variants that showed evidence of association in the Cervigen cohort for known eQTL effects in immune cells, such as CD4 positive regulatory memory T cells, CD4 positive TH1 cells, or naive B cells.

### 2.6 Transcript Analysis

Total RNA was isolated from methanol‐fixed cervical tissue smears (*n* = 303) via guanidinium‐phenol‐chloroform extraction with Trizol reagent (peqGOLD TriFast). From 1 µg RNA of each sample, cDNA was synthesized using the Proto‐Script II First Strand cDNA Synthesis Kit (New England BioLabs). After preamplification of the cDNA samples using pooled DeltaGene assays and the Preamplification Master Mix (Fluidigm), real‐time qPCR was performed on the BioMark HD system (Fluidigm).

To study the GWS variant at the *HLA* locus on chromosome 6, rs9272293, we designed Fluidigm Deltagene assays for 36 candidate genes embedded in this region, together with epithelial cell marker genes *KRT8*, *KRT18,* and *EPCAM*, and housekeeping genes *B2M* and *RPL13A* (Table S6). The assays were mixed with the 2× SsoFast EvaGreen Super‐mix with low ROX (BioRad) to detect gene amplification curves. Two samples without cDNA were included as negative controls. Twenty‐three samples with low gene expression levels (C_T_ above 32) for the epithelial markers *EPCAM*, *KRT8,* or *KRT18* were excluded from further statistical analysis. Normalization of target gene expression data against the housekeeping genes *B2M* and *RPL13A* was carried out with the qBASE+ software (Biogazelle). Outliers were removed from the dataset using the ROUT method on Graphpad Prism v10.1 (Dotmatics).

Genomic DNA from the same samples (*n* = 280) was isolated in a magnetic bead‐based purification process on the M24 SP instrument (Abbott) and subjected to SNP genotyping. For eQTL analysis, the log10 normalized gene expression values of 280 cDNA samples were analyzed for association with the genotypes of the lead variant rs9272293 in matching DNA samples. Genotypes and gene expression levels were investigated under an allelic model in all samples (overall) as well as after stratification based on HPV status (HPV positive or HPV negative sub‐groups). For some genes with low levels of gene expression, an additional Pearson's correlation test was performed in GraphPad Prism v10 to explore whether the genotype correlates with the presence or absence (C_T_ above 32) of gene transcripts (Supplementary Table S7). For the comparison of three groups, ANOVA was applied, and p values were also reported after a linear test for trend. Multiple testing correction threshold (Bonferroni method) was applied as 36 genes were tested to be eQTLs (*α* = 0.05/36 = 1.4 × 10E‐3) and only SNP‐gene pairs passing this threshold were considered to be robust eQTLs. For gene‐gene correlation analysis, Pearson's R was calculated in GraphPad Prism v10.

## 3 Results

### 3.1 GWAS Outcomes

**Figure 1:**
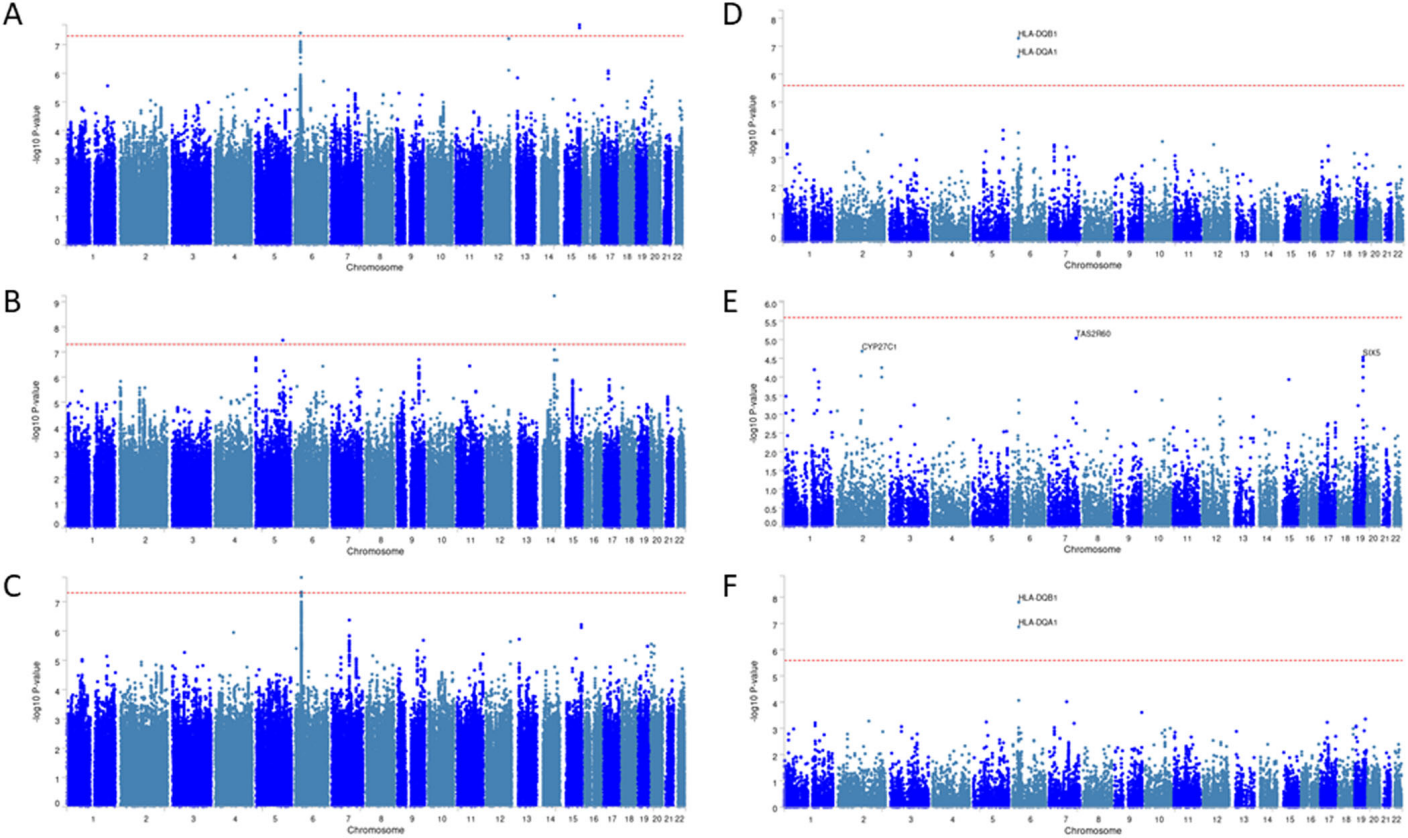
GWAS plots for HPV seropositivity GWASs. −log10 p values from the GWAS summary statistics are plotted on the *y* axis for (A) HPV16 seropositivity, (B) HPV18 seropositivity and (C) HPV16 and/or 18 seropositivity in Manhattan plots. Chromosomes are represented on the *x* axis. The dotted red line is set at the genome‐wide significance level of 5 × 10E‐8. Furthermore, −log10 *p* values after MAGMA gene‐based analysis are shown with Manhattan plots for (D) HPV16 seropositivity, (E) HPV18 seropositivity and (F) HPV16 and/or 18 seropositivity. Since SNPs were mapped to 19,380 protein coding genes, the red line indicates *p* = 0.05/19380 = 2.58 × 10E‐6.

GWAS analysis for HPV16 seropositivity identified three variants at GWS, rs9272293 G > A at 6p21.32 (OR = 0.71 (allele A), SE = 0.06; *p* = 3.93 × 10E‐8) and rs1828768 (T > C) and rs991757 (A > G) at 15q26.2 (OR = 0.69 (allele T), SE = 0.07; *p* = 2.02 × 10E‐8; and OR = 0.69 (allele A), SE = 0.07; *p* = 2.59 × 10E‐8, respectively) (Figure 1A), whereas for HPV18 seropositivity two variants (rs142237244 G > A at 5q31.2 with OR = 4.95 (allele A), SE = 0.29; *p* = 3.43 × 10E‐8; and rs4243652 at 14q24.3 with OR = 3.14 (allele G), SE = 0.19; *p* = 5.84 × 10E‐10) were genome‐wide significant (Figure 1B). For HPV16 and/or HPV18 seropositivity, we found two genome‐wide significant variants (rs9272293 with OR = 0.72 (allele A), SE = 0.06; *p* = 1.49 × 10E‐8 and rs17612669 at 6p21.32 with OR = 0.73 (allele G), SE = 0.06; *p* = 4.64 × 10E‐8) (Figure 1C). In genome‐wide gene‐based analysis in MAGMA, *HLA‐DQA1* and *HLA‐DQB1* were identified as genome‐wide significant regions, corroborating the signals at 6p21.32 (Figure 1D–F). In the gene‐set enrichment analysis in MAGMA, no gene sets were detected with a Bonferroni corrected p‐value below 0.05 in either of the three GWAS data sets (Table S8A). However, in the GENE2FUNC functionality of FUMA, in gene set enrichment analysis, Immunologic signatures (MsigDB c7), 117, 0, and 63 gene sets were identified as enriched in the HPV16, HPV18, and HPV16/18 seropositivity analysis, respectively (Table S8B). All the gene set enrichment results from GENE2FUNC for each of the GWASs are noted in Table S8C. In fine‐mapping analysis using SuSiE [21], only one 95% credible set was identified with the lead variant rs9272293 having the highest PIP of 0.066. This credible set included 889 variants (Table S9A, Figure S3). Fine‐mapping using Rsparsepro [22] identified a single credible set of 814 variants, with the lead variant rs9272293 having the highest PIP of 0.099 (Table S9B).

### 3.2 Cervical Cancer Case‐Control Genotyping

Among the GWS variants, rs9272293 and rs17612669 are moderately linked with *R* ^2^ = 0.45 in 1000G EUR and highly linked in UKB EUR GRCh37 (*R^2^* = 0.89), and the former was taken for TaqMan genotyping due to the significant association with HPV16 only and HPV16/HPV18 in GWAS results. Also, rs991757 and rs1828768 are in high linkage disequilibrium (*R* ^2^ = 0.99 1KG EUR) and the former was taken forward for PCR‐based genotyping. rs142237244 was not genotyped due to its low MAF in Europeans (0.0096 in 1000 G), and rs4243652 had already been described as successfully replicated in our previous cervical cancer case‐control study [15]. We thus forwarded rs9272293 and rs991757 as two independent GWS signals to the validation stage.

We also added further sub‐genome‐wide significant variants to test their potential role in cervical cancer. From 170, 193, and 677 variants, respectively, at *p* < 5 × 10E‐6 (summary statistics provided in Table S1), we filtered 27 variants for further PCR‐based case‐control genotyping in the Cervigen cohort (Figure S2, Table S2).

We genotyped up to 2484 cases and 1226 controls of the Cervigen series (Table 1, Table S10). Four variants were removed due to poor clustering or failing HWE, leaving 23 sub‐genome‐wide variants in addition to the two GWS signals for the association analyses. For these 25 candidate variants, call rates were at least 95% (Table S3). Approximately 10% of all samples were repeated and concordance rates ranged between 83.9% and 100% (Table S3).

**Table 1:** Results after logistic regression analyses from the genetic case‐control study.

Variant ID	rs9272293 (HPV16+, HPV16/18+)				rs17867660 (HPV16+, HPV16/18+)				rs6084436 (HPV16+, HPV16/18+)			
Stratum	Controls	Cases	OR (95% CI)	*p* value	Controls	Cases	OR(95%CI)	*p* value	Controls	Cases	OR (95% CI)	*p* value
**Overall**	1226	2484	0.86 (0.78–0.95)	**0.004**	1073	1310	0.97 (0.87–1.09)	0.604	1063	1292	1.08 (0.86–1.34)	0.515
**Dysplasia**	1226	1373	0.90 (0.80–1.00)	0.055	1073	868	1.05 (0.92–1.19)	0.452	1063	831	1.04 (0.81–1.34)	0.74
LSIL	1226	228	0.93 (0.76–1.13)	0.455	1073	196	0.99 (0.79–1.23)	0.907	1063	189	1.22 (0.81–1.84)	0.331
HSIL	1226	1145	0.89 (0.79–1.00)	0.054	1073	672	1.07 (0.93–1.23)	0.339	1063	642	0.99 (0.76–1.30)	0.961
**Invasive**	1226	1075	0.83 (0.74–0.93)	**0.002**	1073	442	0.83 (0.70–0.97)	**0.02**	1063	461	1.14 (0.85–1.53)	0.383
Adenocarcinoma	1226	186	0.78 (0.62–0.97)	**0.026**	1073	71	0.73 (0.51–1.04)	0.078	1063	74	1.21 (0.65–2.23)	0.55
Squamous carcinoma	1226	698	0.87 (0.76–0.99)	**0.038**	1073	340	0.85 (0.71–1.01)	0.065	1063	364	1.10 (0.79–1.52)	0.583
**Invasive** + **HSIL combined**	1226	2220	0.86 (0.78–0.95)	**0.003**	1073	1114	0.97 (0.86–1.09)	0.582	1063	1103	1.05 (0.84–1.33)	0.663
**HPV16 + ve Overall**	1226	329	0.76 (0.64–0.91)	**0.003**	1073	321	0.95 (0.80–1.14)	0.586	1063	301	1.38 (1.00–1.92)	0.054
HPV16 + ve Invasive + HSIL	1226	308	0.76 (0.64–0.91)	**0.004**	1073	299	0.95 (0.79–1.14)	0.575	1063	286	1.35 (0.96–1.89)	0.084
**HPV18 + ve Overall**	1226	116	0.82 (0.62–1.08)	0.164	1073	121	0.95 (0.72–1.24)	0.7	1063	102	1.49 (0.91–2.45)	0.113
HPV18 + ve Invasive + HSIL	1226	109	0.83 (0.62–1.10)	0.194	1073	113	0.98 (0.74–1.30)	0.882	1063	98	1.39 (0.83–2.33)	0.215
**HPV16/18 + ve Overall**	1226	420	0.78 (0.66–0.91)	**0.002**	1073	413	0.94 (0.80–1.11)	0.485	1063	386	1.41 (1.05–1.90)	**0.023**
HPV16/18 + ve Invasive + HSIL	1226	394	0.77 (0.66–0.91)	**0.002**	1073	386	0.94 (0.79–1.11)	0.445	1063	367	1.36 (1.00–1.85)	0.051
**HPV (other) + ve Overall**	1226	95	0.74 (0.54–1.00)	0.054	1073	90	1.19 (0.88–1.62)	0.256	1063	82	1.08 (0.58–2.00)	0.807
HPV (other) + ve Invasive + HSIL	1226	71	0.77 (0.55–1.10)	0.147	1073	68	1.23 (0.87–1.75)	0.241	1063	63	0.93 (0.44–1.94)	0.837

Variant ID	rs11658042 (HPV16+)				rs742625 (HPV16+)				rs115851441 (HPV18+)			
Stratum	Controls	Cases	OR (95% CI)	*p* value	Controls	Cases	OR (95% CI)	*p* value	Controls	Cases	OR (95% CI)	*p* value
**Overall**	1067	1328	0.95 (0.80–1.13)	0.558	1200	1354	0.53 (0.32–0.88)	**0.014**	1070	1349	0.71 (0.52–0.97)	**0.031**
**Dysplasia**	1067	873	1.00 (0.83–1.21)	0.996	1200	879	0.56 (0.31–0.99)	**0.045**	1070	880	0.73 (0.52–1.03)	0.073
LSIL	1067	199	1.01 (0.74–1.40)	0.929	1200	198	0.73 (0.29–1.88)	0.516	1070	198	0.36 (0.16–0.83)	**0.016**
HSIL	1067	674	1.00 (0.81–1.22)	0.972	1200	681	0.51 (0.26–0.97)	**0.041**	1070	682	0.84 (0.59–1.20)	0.343
**Invasive**	1067	455	0.85 (0.66–1.09)	0.193	1200	475	0.48 (0.23–1.04)	0.063	1070	469	0.69 (0.44–1.06)	0.09
Adenocarcinoma	1067	72	1.48 (0.93–2.35)	0.099	1200	75	0.77 (0.18–3.27)	0.728	1070	75	0.96 (0.42–2.21)	0.926
Squamous carcinoma	1067	352	0.71 (0.53–0.95)	**0.019**	1200	368	0.39 (0.15–0.99)	**0.048**	1070	363	0.69 (0.43–1.12)	0.13
**Invasive** + **HSIL combined**	1067	1129	0.94 (0.78–1.12)	0.487	1200	1156	0.50 (0.29–0.85)	**0.011**	1070	1151	0.78 (0.57–1.06)	0.113
**HPV16 + ve Overall**	1067	330	0.87 (0.66–1.15)	0.32	1200	350	0.49 (0.21–1.17)	0.109	1070	345	0.87 (0.55–1.36)	0.537
HPV16 + ve Invasive + HSIL	1067	308	0.88 (0.67–1.17)	0.387	1200	328	0.44 (0.17–1.12)	0.084	1070	323	0.89 (0.56–1.41)	0.619
**HPV18 + ve Overall**	1067	121	0.94 (0.62–1.42)	0.762	1200	121	.	.	1070	120	0.70 (0.32–1.51)	0.362
HPV18 + ve Invasive + HSIL	1067	113	1.02 (0.67–1.55)	0.937	1200	113	.	.	1070	112	0.53 (0.22–1.32)	0.173
**HPV16/18 + ve Overall**	1067	422	0.86 (0.67–1.11)	0.249	1200	442	0.39 (0.16–0.92)	**0.032**	1070	436	0.85 (0.56–1.29)	0.445
HPV16/18 + ve Invasive + HSIL	1067	395	0.88 (0.68–1.14)	0.351	1200	415	0.34 (0.14–0.88)	**0.026**	1070	409	0.82 (0.53–1.26)	0.362
**HPV(other) + ve Overall**	1067	91	0.80 (0.49–1.32)	0.383	1200	91	0.31 (0.04–2.31)	0.255	1070	91	0.39 (0.12–1.25)	0.113
HPV(other) + ve Invasive + HSIL	1067	69	0.78 (0.44–1.37)	0.384	1200	69	0.42 (0.06–3.07)	0.389	1070	69	0.17 (0.02–1.24)	0.081

Variant ID	rs114272671 (HPV18+)				rs12207703 (HPV18+)				rs72808738 (HPV18+)			
Stratum	Controls	Cases	OR (95% CI)	*p* value	Controls	Cases	OR (95% CI)	*p* value	Controls	Cases	OR (95% CI)	*p* value
**Overall**	1203	1349	0.89 (0.61–1.30)	0.543	1071	1316	0.95 (0.83–1.09)	0.464	1071	1355	0.94 (0.66–1.34)	0.743
**Dysplasia**	1203	878	1.05 (0.70–1.57)	0.823	1071	867	1.01 (0.87–1.17)	0.942	1071	880	0.92 (0.62–1.36)	0.674
LSIL	1203	199	0.99 (0.48–2.03)	0.975	1071	195	1.09 (0.86–1.40)	0.474	1071	199	1.04 (0.55–1.98)	0.909
HSIL	1203	679	1.06 (0.69–1.65)	0.779	1071	672	0.98 (0.83–1.15)	0.804	1071	681	0.88 (0.58–1.36)	0.576
**Invasive**	1203	471	0.59 (0.32–1.09)	0.095	1071	449	0.85 (0.71–1.03)	0.09	1071	475	0.99 (0.62–1.58)	0.963
Adenocarcinoma	1203	74	1.51 (0.59–3.90)	0.392	1071	72	0.90 (0.6–1.36)	0.627	1071	75	2.80 (1.44–5.41)	**0.002**
Squamous carcinoma	1203	365	0.41 (0.18–0.90)	**0.027**	1071	345	0.78 (0.64–0.97)	**0.022**	1071	367	0.62 (0.33–1.15)	0.127
**Invasive** + **HSIL combined**	1203	1150	0.87 (0.59–1.30)	0.499	1071	1121	0.93 (0.81–1.07)	0.285	1071	1156	0.93 (0.64–1.34)	0.684
**HPV16 + ve Overall**	1203	347	0.43 (0.19–0.95)	**0.038**	1071	325	0.99 (0.80–1.21)	0.903	1071	349	0.76 (0.43–1.35)	0.352
HPV16 + ve Invasive + HSIL	1203	325	0.46 (0.21–1.02)	0.056	1071	304	0.95 (0.77–1.18)	0.666	1071	327	0.81 (0.46–1.44)	0.473
**HPV18 + ve Overall**	1203	121	0.35 (0.08–1.46)	0.149	1071	118	0.88 (0.64–1.22)	0.445	1071	120	1.25 (0.60–2.62)	0.552
HPV18 + ve Invasive + HSIL	1203	113	0.19 (0.03–1.36)	0.098	1071	111	0.88 (0.63–1.23)	0.471	1071	112	1.17 (0.54–2.56)	0.689
**HPV16/18 + ve Overall**	1203	439	0.44 (0.21–0.89)	**0.023**	1071	417	0.92 (0.77–1.12)	0.412	1071	441	0.90 (0.55–1.47)	0.667
HPV16/18 + ve Invasive + HSIL	1203	412	0.41 (0.20–0.88)	**0.021**	1071	391	0.90 (0.74–1.09)	0.278	1071	414	0.91 (0.55–1.50)	0.713
**HPV (other) + ve Overall**	1203	91	0.71 (0.22–2.32)	0.573	1071	88	1.15 (0.81–1.62)	0.445	1071	90	0.63 (0.20–2.00)	0.433
HPV (other) + ve Invasive + HSIL	1203	69	0.62 (0.15–2.61)	0.517	1071	67	1.12 (0.75–1.67)	0.57	1071	68	0.56 (0.14–2.27)	0.413

Variant ID	rs76710445 (HPV18+)				rs79316639 (HPV18+)				rs13288372 (HPV16/18+)			
Stratum	Controls	Cases	OR (95% CI)	*p* value	Controls	Cases	OR (95% CI)	*p* value	Controls	Cases	OR(95%CI)	*p* value
**Overall**	1202	1352	0.74 (0.51–1.05)	0.093	906	1349	1.54 (1.01–2.34)	**0.043**	1064	1310	1.15 (1.01–1.31)	**0.032**
**Dysplasia**	1202	879	0.64 (0.42–0.98)	**0.04**	906	881	1.57 (1.01–2.46)	**0.046**	1064	860	1.18 (1.02–1.35)	**0.024**
LSIL	1202	198	0.25 (0.08–0.81)	**0.021**	906	199	1.14 (0.53–2.45)	0.745	1064	194	0.98 (0.77–1.25)	0.863
HSIL	1202	681	0.76 (0.49–1.17)	0.214	906	682	1.70 (1.07–2.70)	**0.025**	1064	666	1.24 (1.06–1.44)	**0.006**
**Invasive**	1202	473	0.92 (0.57–1.47)	0.716	906	468	1.46 (0.86–2.48)	0.166	1064	450	1.10 (0.92–1.31)	0.28
Adenocarcinoma	1202	74	1.19 (0.46–3.05)	0.717	906	74	1.89 (0.74–4.83)	0.182	1064	73	1.10 (0.76–1.60)	0.602
Squamous carcinoma	1202	366	0.90 (0.53–1.52)	0.69	906	361	1.41 (0.79–2.52)	0.242	1064	345	1.12 (0.92–1.35)	0.249
**Invasive** + **HSIL combined**	1202	1154	0.82 (0.57–1.18)	0.29	906	1150	1.61 (1.05–2.46)	**0.029**	1064	1116	1.18 (1.04–1.35)	**0.013**
**HPV16 + ve Overall**	1202	350	0.89 (0.52–1.52)	0.669	906	346	1.53 (0.88–2.68)	0.135	1064	326	1.07 (0.88–1.30)	0.478
HPV16 + ve Invasive + HSIL	1202	328	0.90 (0.52–1.55)	0.698	906	324	1.55 (0.88–2.73)	0.132	1064	304	1.11 (0.91–1.35)	0.32
**HPV18 + ve Overall**	1202	121	0.86 (0.36–2.02)	0.723	906	121	1.17 (0.46–2.97)	0.746	1064	120	1.04 (0.77–1.41)	0.784
HPV18 + ve Invasive + HSIL	1202	113	0.92 (0.39–2.17)	0.85	906	113	1.25 (0.49–3.17)	0.642	1064	112	1.09 (0.81–1.49)	0.561
**HPV16/18 + ve Overall**	1202	442	0.86 (0.53–1.41)	0.549	906	438	1.41 (0.82–2.40)	0.212	1064	418	1.07 (0.89–1.27)	0.49
HPV16/18 + ve Invasive + HSIL	1202	415	0.88 (0.53–1.45)	0.603	906	411	1.43 (0.83–2.46)	0.197	1064	391	1.10 (0.91–1.31)	0.329
**HPV (other) + ve overall**	1202	90	0.57 (0.17–1.84)	0.343	906	91	0.93 (0.29–3.02)	0.91	1064	89	1.14 (0.82–1.59)	0.444
HPV (other) + ve Invasive + HSIL	1202	68	0.76 (0.23–2.47)	0.646	906	69	1.23 (0.38–3.95)	0.733	1064	67	0.96 (0.65–1.43)	0.841

*Note:* Cervical intraepithelial neoplasia was separated into LSIL/low‐grade (CIN1 + CIN2 < 30 years) and HSIL/high‐grade (CIN2 ≥ 30 years + CIN3) subgroups. Invasive cervical cancer was further divided into squamous epithelial cell carcinoma and adenocarcinoma. High‐risk dysplasia (CIN2 ≥ 30years + CIN3) and invasive cancer were also combined in joint analysis. HSIL and invasive cancers were further stratified by HPV status (HPV 16, HPV18, HPV16/18 and other hrHPV). All groups were compared against cancer‐ and dysplasia‐free controls (with unknown serostatus). *p* values below 0.05 are marked in bold.

Abbreviations: CI, 95% confidence interval; OR, odds ratio for minor allele; *p*, *p* value from logistic regression analysis.

In logistic regression analyses, rs9272293 representing the GWS signal from our HPV16 and HPV16/18 GWASs at 6p21.32, was associated with overall cervical disease (OR = 0.86, *p* = 0.004, 95% CI = 0.78–0.95) (Table 1). When stratified for disease severity, we found rs9272293 to be associated with invasive cervical cancer (OR = 0.83, *p* = 0.002, 95% CI = 0.74–0.93), cervical adenocarcinoma (OR = 0.78, *p* = 0.026, 95% CI = 0.62–0.97) and squamous cervical carcinoma (OR = 0.87, *p* = 0.038, 95% CI = 0.76–0.99). The variant associated with HPV positive overall cervical disease (OR = 0.83, *p* = 0.001, 95% CI = 0.74–0.93), and in stratified analysis, with HPV16+ , HPV16/18+ overall disease and in a combined analysis of HPV16+ or HPV16/18 + HSIL and invasive cancers (Table 1).

Among the sub‐genome‐wide significant candidates, four further variants showed evidence of association with overall cervical disease: rs742625 at 20p12.1 (HPV16+, HPV16/18+), rs79316639 at 2p25.2 (HPV18+), rs115851441 at 5p15.33 (HPV18+), and rs13288372 at 9q34.11 (HPV16/18+ ) (Table 1 and Table S10). When analyses were restricted to invasive cervical cancer or specific histology groups, rs17867660 from the HPV16 seropositivity GWAS at 7q21.13 showed some evidence of association with overall invasive cancer (Table 1). Two other variants from the HPV16 seropositivity GWAS, rs11658042 at 17q12 and rs742625 at 20p12.1, as well as two variants from the HPV18 seropositivity GWAS, rs114272671 at 5q31.3 and rs12207703 at 6p24.1, showed evidence of association with squamous cervical carcinoma. One variant, rs72808738 from the HPV18 seropositivity GWAS at 5q31.3, was associated with adenocarcinoma of the cervix (Table 1).

In stratified analyses of dysplasia, rs79316639 and rs13288372 showed evidence of association with cervical dysplasia overall, high grade cervical dysplasia, and in combined analysis, with high grade cervical dysplasia and invasive cervical cancer (Table 1). By contrast, rs76710445 and rs115851441 (both from HPV18 + GWAS) were also associated with low grade cervical dysplasias.

When stratified by HPV type (Table 1), rs114272671 was associated with HPV16+ cervical disease, and two variants rs6084436 and rs742625 were associated with HPV16/18+ cervical disease, whereas no variant was selectively associated with HPV18+ cervical disease in our analysis. However, sample numbers were the smallest for HPV18+ status. In the combined analysis of HPV16 and/or 18 positive high‐grade cervical dysplasia and invasive cervical cancer samples, we found further evidence of significant association with rs114272671 and rs742625.

Taken together, 12 out of the 25 candidate variants from the seropositivity GWAS showed some evidence of association with cervical cancer, either overall (5 variants) or only in subgroups (7 variants) (Table 1).

### 3.3 Bioinformatic Annotation

Up to 10 genes in close physical proximity to the top variants within 2Mbp (+/− 1Mbp of the variant) were noted as putative candidate causal genes, as the levels of these genes may be influenced by the SNP genotype (Table S4A). The most significant variant, rs9272293, was reported to be a robust eQTL for multiple genes at the HLA region in whole blood in GTEx v8 (*HLA‐DQB1* with *p* = 1.65 × 10E‐97 and *HLA‐DQB2* with *p* = 3.07 × 10E‐89, among others, Figure S3). Amongst our sub‐GWS variants that were genotyped, rs17867660 was found to be an eQTL in GTEx whole blood for *GTPBP10* at 7q21.13 (*p* = 3.3 × 10E‐8, normalized effect size 0.13) and also in the eQTLGen consortium whole blood (*p* = 1.73 × 10E‐44, *z*‐score = 13.99) (Table S4A, Table S5). rs11658042 was an eQTL for *SLFN5* at 17q12 in GTEx whole blood (*p* = 2.0 × 10E‐11, normalized effect size 0.23) and in the eQTLGen consortium for *SLFN5, SLFN12L, SLFN13, SLFN11, RP11‐47L3.1, RP11‐799D4.4*, and *RP11‐1094M14.5* (Figure S4, Table S4A, Table S5). The SNP rs12207703 was an eQTL for *TBC1D7* in whole blood in the eQTLGen consortium data (*p* = 1.3 × 10E‐9, *z*‐score = 6.07). None of the twelve significant variants were reported to be direct eQTLs in immune cell types accessed via DICE.

In bioinformatic analysis, we predicted allele‐specific transcription factor binding sites using HaploReg v4.2, RegulomeDB, and the PERFECTOS‐APE webtool for the 12 variants that showed evidence of association in at least one subgroup (scores provided in Table S4B, Table S11, Table S12, Table S13). We also submitted the top 12 variants to FORGEdb for annotation, however, the results were largely similar to individual annotations via HaploReg v4.2, GTEx, eQTLGen and RegulomeDB (Table S14). The chromatin status in HeLa cells was quiescent or weakly repressed polycomb for 11 out of 12 submitted variants according to the Regulome database, except for rs13288372, which appears to be in an enhancer element (Table S4A). The annotations for all linked variants at *R* ^2^ ≥ 0.8 were also generated using HaploReg v4.2 and RegulomeDB (Table S11, Table S12).

### 3.4 Gene Expression Analysis

**Figure 2:**
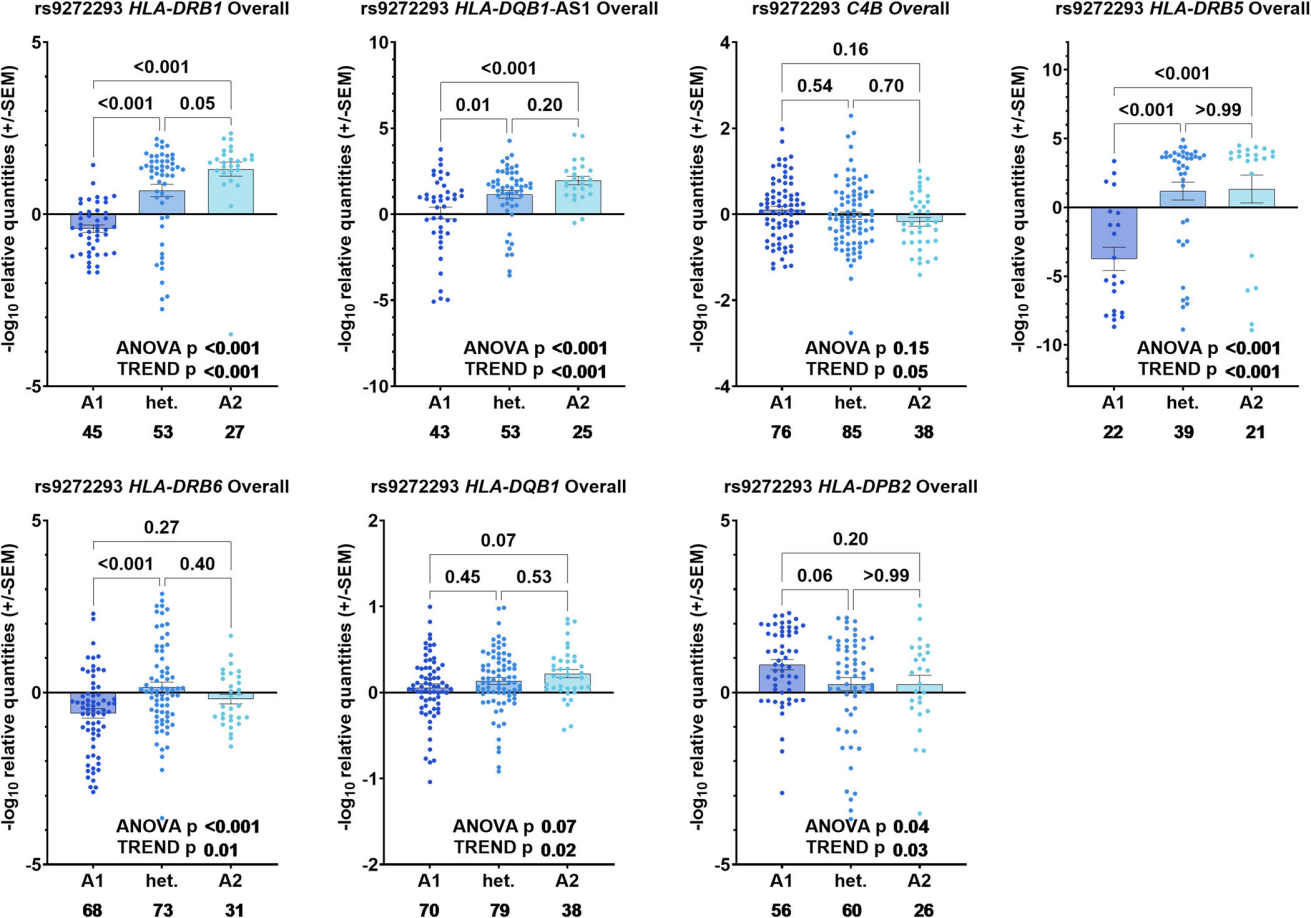
eQTL analysis for rs9272293 in all cervical tissues. −log10 relative quantities (+/−SEM) are shown on the *y* axis, together with genotypes on the *x* axis (A1 = common homozygous genotype GG, het. = heterozygous genotype GA, A2 = rare homozygous genotype AA). Sample numbers per group are written below the respective bars on the *x* axis. *p* values are indicated after ANOVA was performed between three groups followed by a linear trend test, with the common genotype as the control.

**Figure 3:**
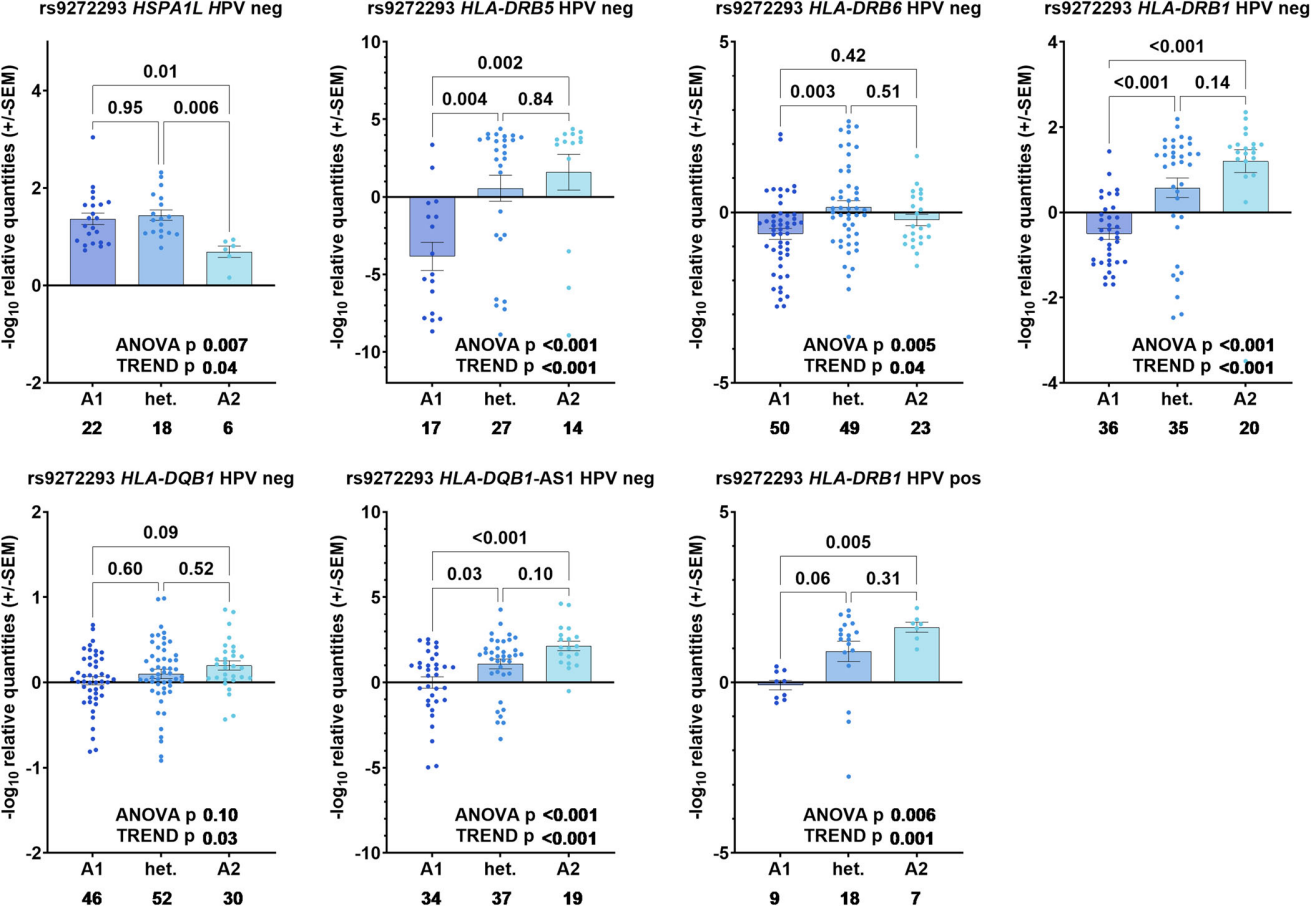
eQTL analysis for rs9272293 in HPV negative and HPV positive cervical tissues. −log10 relative quantities (+/− SEM) are shown on the *y* axis, together with genotypes on the *x* axis (A1 = common homozygous genotype GG, het. = heterozygous genotype GA, A2 = rare homozygous genotype AA). Sample numbers per group are written below the respective bars on the *x* axis. *p* values are indicated after ANOVA was performed between three groups followed by a linear trend test, with the common genotype as the control.

We performed eQTL analysis in our series of cervical tissue scrapes (*n* = 280) to investigate how the genotype of the most significant variant rs9272293 at 6p21.32 can affect the expression of the neighboring genes at this locus. We found rs9272293 to be an eQTL for multiple genes among all samples tested: *HLA‐DRB1*, *HLA‐DQB1‐AS1*, *C4B*, *HLA‐DRB5*, *HLA‐DRB6*, *HLA‐DQB1*, and *HLA‐DPB2* (Figure 2). Transcript levels of *HLA‐DRB1* (*P*_Trend_ < 0.001), *HLA‐DQB1‐AS1* (*P*_Trend_ < 0.001), *HLA‐DRB5* (*P*_Trend_ < 0.001), *HLA‐DRB6* (*P*_Trend_ = 0.01), and *HLA‐DQB1* (*P*_Trend_ = 0.02) were increased in the presence of the rare allele A that was protective in our association analyses (Figure 2). Transcript levels of *C4B* (*P*_Trend_ = 0.05) and *HLA‐DPB2* (*P*_Trend_ = 0.03) appeared decreased with the rare homozygous genotype (Figure 2). The eQTL effects for rs9272293 with *HLA‐DQB1‐AS1*, *HLA‐DRB5*, *HLA‐DRB6*, and *HLA‐DQB1* were mainly observed in HPV negative (noncancerous/dysplastic) samples (*HLA‐DQB1‐AS1*: *P*_Trend_ < 0.001, *HLA‐DRB5*: *P*_Trend_ < 0.001, *HLA‐DRB6*: *P*_Trend_ = 0.04, and *HLA‐DQB1*: *P*_Trend_ = 0.03, *HSPA1L*: *P*_Trend_ = 0.04 (Figure 3), whereas rs9272293 remained an eQTL for *HLA‐DRB1* in both HPV positive (*P*_Trend_ < 0.001) and HPV negative samples (*P*_Trend_ = 0.001).

**Figure 4:**
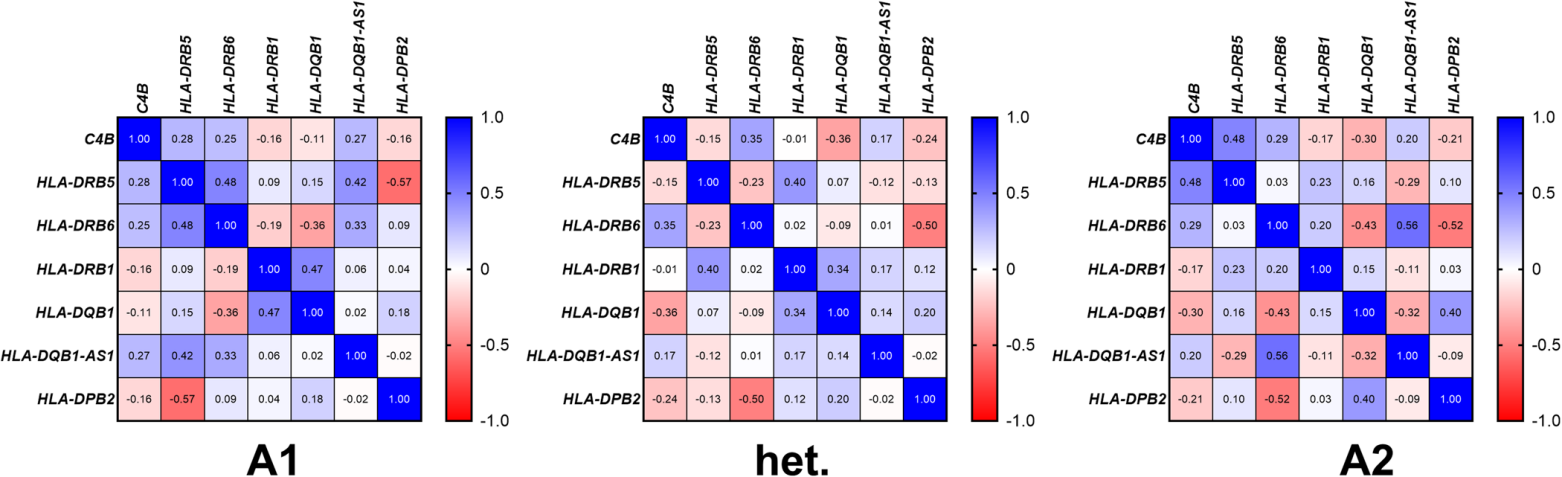
Correlation analysis for genes that are eQTLs for rs9272293. Pearson correlation R values are shown between genes that showed evidence to be eQTLs for rs9272293. Panels from left to right indicate the genotype of rs9272293 (A1 = common homozygous genotype GG, het. = heterozygous genotype GA, A2 = rare homozygous genotype AA). Negative correlation (*R* = −1) is shown in deep red color, whereas positive correlation (*R* = 1) is shown in deep blue color. Missing values are represented with a black X through the white box.

For the eight eQTL genes of rs9272293, a pairwise gene‐gene correlation analysis was performed to examine the influence of genotype on coordinated gene expression. We found evidence for a differential correlation between some of these genes under the three genotypes of rs9272293, for example, for the pair *HLA‐DPB2*/*HLA‐DRB5* (Figure 4). For gene transcripts that were very low expressed, Pearson correlation analysis revealed that rs9272293 genotype may additionally associate with the detection of further genes (CT ≤ 32) beyond the eQTL genes mentioned above (Table S7).

## 4 Discussion

Genome‐wide association studies for cervical cancer have identified multiple susceptibility variants on four chromosomal regions, most of them arising at the HLA locus [6, 7, 8, 9, 10, 11, 12]. A persistent infection with high risk HPV types is found in almost all cervical cancers [4]. Given this prominent role of HPV infection and the evidence for HLA‐mediated cervical cancer risk, HPV seropositivity GWASs can provide insight into further HPV type specific genetic risk factors for cervical cancer [15, 16, 28].

In this study, we aimed to test variants from HPV seropositivity GWASs for their role in cervical cancer risk. Out of 25 candidates, twelve independent signals showed evidence of association in our case‐control cohort for cervical cancer and dysplasia in at least one of the subgroups, with five of them being associated with overall cervical disease. Variant rs9272293 at 6p21.32 was the lead variant from the GWAS for both HPV16 as well as HPV16/18 serostatus and had the highest PIP in a credible set in fine‐mapping analysis using SuSiE and Rsparsepro. rs9272293 associated mainly with HPV16‐positive cancers, consistent with the initial seropositivity GWAS since the association for the signal represented by rs9272293 was largely driven by HPV16 while HPV18 seropositive GWAS alone did not identify significant associations at 6p21.32‐33 (HLA region) at GWS. This variant is not linked with previously reported CC GWAS susceptibility loci in this region [6] and thus represents an independent novel signal. We additionally found eQTL evidence for this SNP, with the rare protective allele associating with increased transcript levels of *HLA‐DQB1, HLA‐DQB1‐AS1*, *HLA‐DRB1*, *HLA‐DRB5* and *HLA‐DRB6* in cervical epithelial cells. As some of these eQTL effects disappeared in HPV positive tissue samples, HPV infection may counteract the protective effect of the rare allele. It is possible that rs9272293 modifies immune response through regulation of MHC class II genes. Variants at *HLA‐DQB1* have previously been associated with an increased risk of cervical disease [29, 30, 31] and increased *HLA‐DQB1* may act as a biomarker for cervical cancer, with its expression being associated with an increased immune response to cervical tumors [32]. The novel lncRNA *HLA‐DQB1‐AS1* was shown to be associated with a GWAS SNP (rs2647046) for Hepatitis‐B associated hepatocellular carcinoma, and has been nominated as a potential oncogene [33, 34], however, no association with cervical cancer has been reported thus far. Expression of *HLA‐DRB* genes also have been associated with cervical cancer risk [35, 36, 37], and induction of these gene products may contribute to exogenous antigen presentation on cervical epithelial cells and activation of T‐helper cells. On the other hand, the protective rare allele of rs9272293 also may be associated with decreased levels of *C4B* and *HLA‐DPB2*. A previous study showed increased levels of *C4B* in cervical carcinoma patients [38] and higher *C4B* levels were associated with decreased survival in the TCGA cervical cancer dataset (https://www.proteinatlas.org/ENSG00000224389-C4B/pathology/cervical+cancer/CESC). Other independent risk variants for CC susceptibility have been reported at *HLA‐DPB2* previously [39, 40] but no clear role of this gene in cervical pathogenesis has been reported to date.

Another HPV16 seropositivity variant that was genome‐wide significant in the initial GWAS, rs991757, did not associate with cervical cancer in the subsequent screening. It is possible that not all of the variants that mediate HPV infection are equally relevant for immune escape and carcinogenesis. Alternatively, our study power was not sufficient to detect a disease‐causing effect, or it could have been a false positive. However, we identified potential associations with cervical cancer for 11 out of 23 additional candidate variants that had been sub‐genome‐wide significant in the initial seropositivity GWAS, with four of them being associated with overall cervical disease and seven with cervical disease subgroups. Among the four variants associated with overall cervical cancer, rs742625 had arisen from the HPV16‐seropositivity GWAS, rs115851441 and rs79316639 from the HPV18‐seropositivity GWAS, and rs13288372 came from the combined HPV16/HPV18 seropositivity GWAS.

Since we did not functionally test the effects of these variants or test them to be eQTLs in our cervical epithelial cohort, we can only speculate on the possible nearest candidate genes that can underlie these signals. rs742625 is located upstream of *SNX5* and *SNORD17*. *SNX5* encodes sorting nexin 5, an endosomal protein involved in viral replication and entry and virus‐induced autophagy [41] and may play an oncogenic role in SCC of head and neck [42] by modulating the degradation of oncogenic proteins such as c‐Myc and Cyclin‐E1 whereas *SNORD17* encodes a small nucleolar RNA that is reported to inactivate p53 and enhance disease progression in hepatocellular carcinoma [43], another virally‐induced cancer.

rs115851441 on 5p15.33 is located close to *hsa‐miR‐4277* and *lnc‐NDUFS6‐3* (361 bp 5′ of *CTD‐2587M23.1)*. This signal is distant and not correlated with previously reported CC susceptibility variants at *CLPTM1L* [11] or an oropharyngeal cancer susceptibility variant (rs10462706) [44] on 5p15.33. rs79316639 at 2p25.2 is located next to the *SRY‐related HMG‐box gene 11* (*SOX11*) gene. *SOX11* is a transcription factor whose expression is associated with HPV status and is downregulated in high‐grade cervical dysplasia and cervical cancer via hypermethylation of its promotor region [45]. rs13288372 is located at *Neuronal Calcium Sensor 1* (*NCS1*) (9q34.11) that encodes a regulator of calcium‐dependent, G protein‐coupled receptor phosphorylation in neurons. *NCS1* overexpression promotes migration and invasion in breast cancer [46], and *NCS1* is an unfavorable prognostic marker in kidney, breast and endometrial cancer (https://www.proteinatlas.org/ENSG00000107130-NCS1).

Among those variants potentially associated with cervical cancer subgroups, the minor allele of rs11658042 at 17q12 was found to be associated with a decreased risk of squamous carcinoma in our cohort. This variant is a reported eQTL for *SLFN5* in whole blood in GTEx v8, with the rare allele increasing levels of *SLFN5*. *SLFN5* encodes Schlafen 5, a p53 target protein, whose role as an antiviral restriction factor in suppressing viral transcription of herpes simplex virus [47] and human immunodeficiency virus (HIV) has been reported previously [48]. The levels of *SLFN5* were shown to be lower in human papillomavirus E5 positive murine dendritic D2.4 cells, indicating a viral response to decrease protective protein levels [49]. *SLFN5* is also reported to play a role in epithelial‐mesenchymal transformation in breast cancer [50], among others [51]. This signal is independent of the previously known CC‐associated GWAS signal on 17q12 (rs8067378, at *GSDMB*) [8].

We investigated whether some of the variants identified here would replicate in other published case‐control datasets for cervical cancer. The lead GWAS variant rs9272293 (HPV16 +,HPV16/18 + ) associated with cervical cancer in both the FinnGen R8 (*p* = 7.2 × 10E‐4) and KoGES PheWeb (*p* = 2.3 × 10E‐2) biobanks, and the linked lead SNP rs17612669 had supportive evidence for association in the Japanese Biobank (*p* = 4.50 × 10E‐2). There was also supportive evidence for some variants below genome‐wide significance, such as rs11658042 (17q12, FinnGen R8 *p* = 3.3 × 10E‐3), rs17867660 (7q21.13, FinnGen R8 *p* = 3.3 × 10E‐2), rs79316639 (2p25.2, UK Biobank *p* = 1.2 × 10E‐3) and rs742625 (20p12.1, KOGES PheWeb *p* = 1.6 × 10E‐2), to be associated with cervical cancer in available GWAS summary statistics from national biobanks, though these biobank data did not allow for stratification by HPV status.

Our study shows that genetic determinants of seropositivity can be relevant in understanding genetic risk in the case of virus‐driven cancers. Cervical cancer is not the only HPV‐driven cancer, and HPV seropositivity variants may be interesting for determining the risk for head and neck cancers and anogenital cancers as well. The first GWAS for HPV seropositivity [14] also reported on replication genotyping in a Latin‐American cohort of head and neck cancer and found supportive evidence for the lead SNP. We further investigated whether the variants arising from the current study are relevant in other published data sets of likely HPV‐associated cancers. rs9272293 associated with vulva cancer (*p* = 1.2 × 10E‐4, *ß *= 0.35) and anal cancer (*p* = 6.6 × 10E‐4, *ß *= 0.46) in FinnGen R8, rs17612669 associated with lung cancer (*p* = 2.5 × 10E‐7) in a meta‐analysis of the KOGES and biobank Japan, rs11658042 and rs17867660 associated with oropharyngeal cancer (*p* = 6.03 × 10E‐3, *ß *= 0.38; *p* = 4.3 × 10E‐2, *ß* = −0.15) in the UK biobank, rs79316639 associated with rectal cancer (*p* = 1.1 × 10E‐2, *ß *= 0.41) in FinnGen R8 and rs742625 associated with laryngeal cancer (*p* = 8.9 × 10E‐3, *ß *= 0.71) in the UK biobank. These observations invite follow‐up investigations for validation in larger cohorts or meta‐analyses with further HPV seropositivity summary statistics, and functional analysis of the proposed risk variants.

Although our findings cast light on the importance of the germline interaction between HPV and cervical cancer, this study has limitations. Our initial selection of HPV associated variants was based on serostatus, not seroreactivity. This was due to sample size limitations in the original UK Biobank GWAS, yet could induce bias in the genetic associations due to behavioral differences (and not genetic alterations) driving HPV status. We also note that our sample size for HPV18‐seropositive individuals was far smaller than for HPV16, and it contained a substantial portion of HPV16/HPV18 double positives. Our association results were thus largely driven by HPV16 seropositivity. While the different design of the HPV18 seropositivity GWAS (including HPV16/HPV18 double positives in the cases and HPV16+/HPV18− as well as HPV16‐/HPV18− in the controls) was for the purposes of statistical power, the lack of signals identified at chromosome 6 as compared to the HPV any seropositivity GWAS (HPV16+ and/or HPV18+ vs. no HPV detected) would indicate that the inclusion of HPV16+/HPV18− samples in the controls of the former GWAS diluted the effects. However, this would not mean that signals arising in the HPV16 and the HPV any GWAS analyses are irrelevant for HPV18 seropositivity. Larger sample sizes with HPV18 only seropositivity may help overcome this limitation of our current study.

Although we confirmed a nominally significant association with cancer risk for many of the HPV seropositivity GWAS signals, others were not associated with cancer. This could be due to the relatively small case‐control series combined with small effect sizes, or due to the lack of a true association. We tested only common variants in this study. Additionally, the genotyped variants may not underlie the association and may act as proxies for the true causal variants that remain to be determined in fine‐mapping studies. At present there are no available resources for such fine‐mapping or eQTL colocalization studies in the cervix, that might provide stronger evidence to identify causal risk loci. Our genetic study was limited to participants of European ancestry and this invites validation studies in larger, diverse cohorts in the future.

Identifying genetic risk variants that act as modulators of this infection‐induced cancer may bring us closer to understanding the molecular mechanisms underlying invasive cervical disease and eventually lead to new strategies in prevention and treatment. Chronic HPV infection is a known risk factor for cervical cancer, and the genetic susceptibility underlying an HPV infection can help inform risk for cervical cancer, as well as for other HPV‐associated cancers. Such variants mediating infection and cancer risk may have pleiotropic effects and are worthy of further investigation.

## 5 Conclusion

Our study corroborates the notion that the shared genetic risk between HPV seropositivity and invasive cervical disease can guide the identification of novel cervical cancer susceptibility loci. Larger GWASs on HPV seropositivity and seroreactivity as well as longitudinal studies on persistent HPV infection risk will be helpful in delineating the genetic risk factors underlying cervical cancer, as well as other HPV‐induced cancers.

## Author Contributions

**Theresa Beckhaus:** formal analysis, investigation, writing–original draft. **Linda Kachuri, Taishi Connor Nakase, Peter Schürmann, Rieke Eisenblätter, Maya Geerts, Tracy A O'Mara,** and **Stephan Francis:** formal analysis, investigation, writing–review and editing. **Gerd Böhmer, Hans‐Georg Strauβ, Christine Hirchenhain, Monika Schmidmayr, Florian Müller, Peter A. Fasching, Alexander Luyten, Norman Häfner, Matthias Jentschke,** and **Peter Hillemanns:** resources, data curation, writing–review and editing. **John S. Witte:** resources, supervision, writing–review and editing. **Thilo Dörk:** data curation, funding acquisition, resources, supervision, writing–original draft. **Dhanya Ramachandran:** formal analysis, investigation, funding acquisition, resources, supervision, visualization, writing–original draft.

## Ethics Statement

The Ethics committee of Hannover Medical School approved this study (votes 441 and 10737).

## Consent

Written and informed consent was collected from each patient that participated in this study. GWAS analyses were conducted with approved access to UK Biobank data under application numbers 14105 and 25331.

## Conflicts of Interest

The authors declare no conflicts of interest.

## Supporting information

Supporting information.

Supporting information.

Supporting information.

Supporting information.

Supporting information.

Supporting information.

Supporting information.

Supporting information.

Supporting information.

Supporting information.

Supporting information.

Supporting information.

Supporting information.

Supporting information.

Supporting information.

Supporting information.

## Data Availability

The data that support the findings of this study are available from the corresponding authors upon request.
