# Supplementary material for: Genome‐Wide Association Analyses of HPV16 and HPV18 Seropositivity Identify Susceptibility Loci for Cervical Cancer
Source: J Med Virol. 2025 Jan 31;97(2):e70195. doi: 10.1002/jmv.70195 (PMC11786146; doi:10.1002/jmv.70195)

Supplementary Figure 1

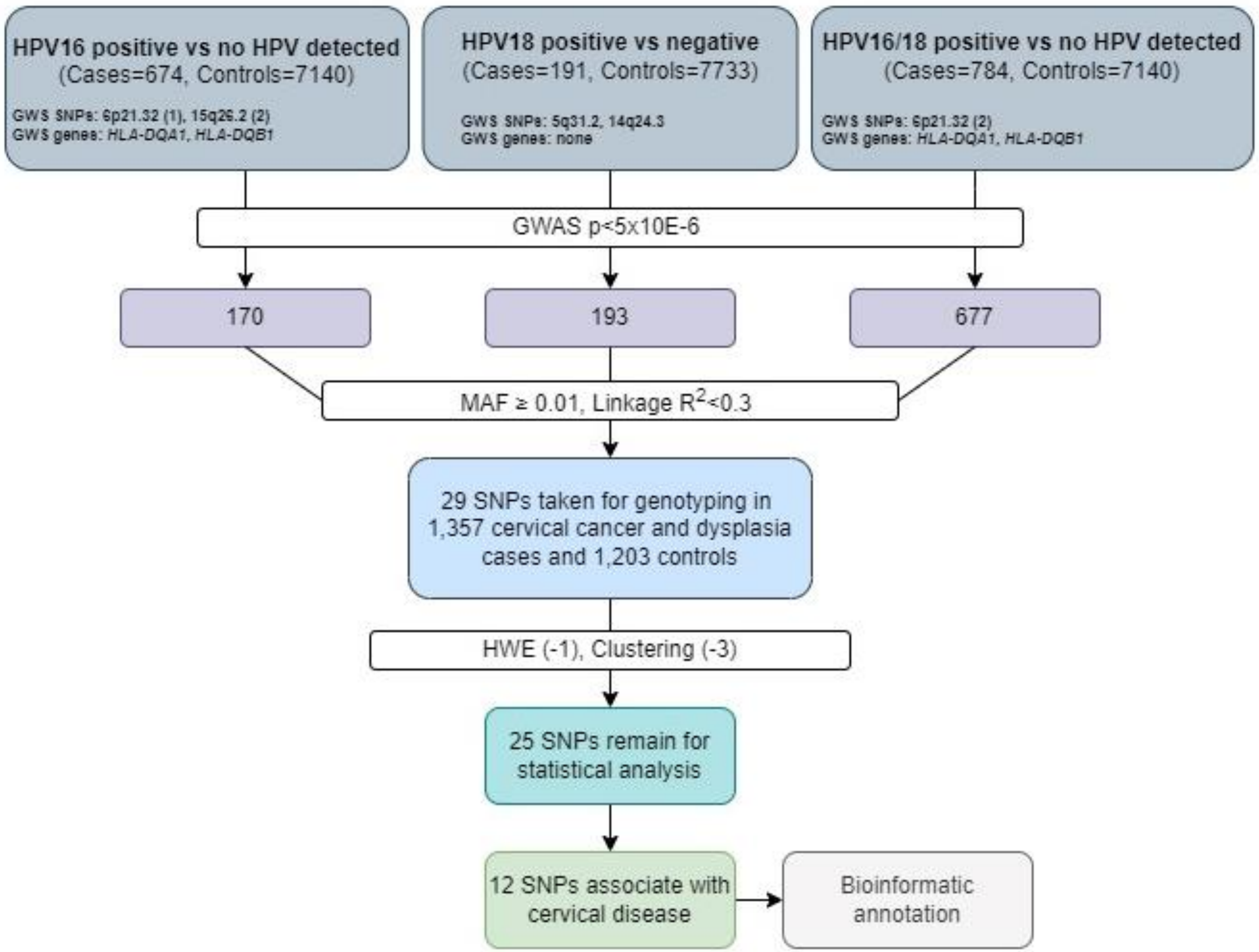

Supplementary Figure 2A

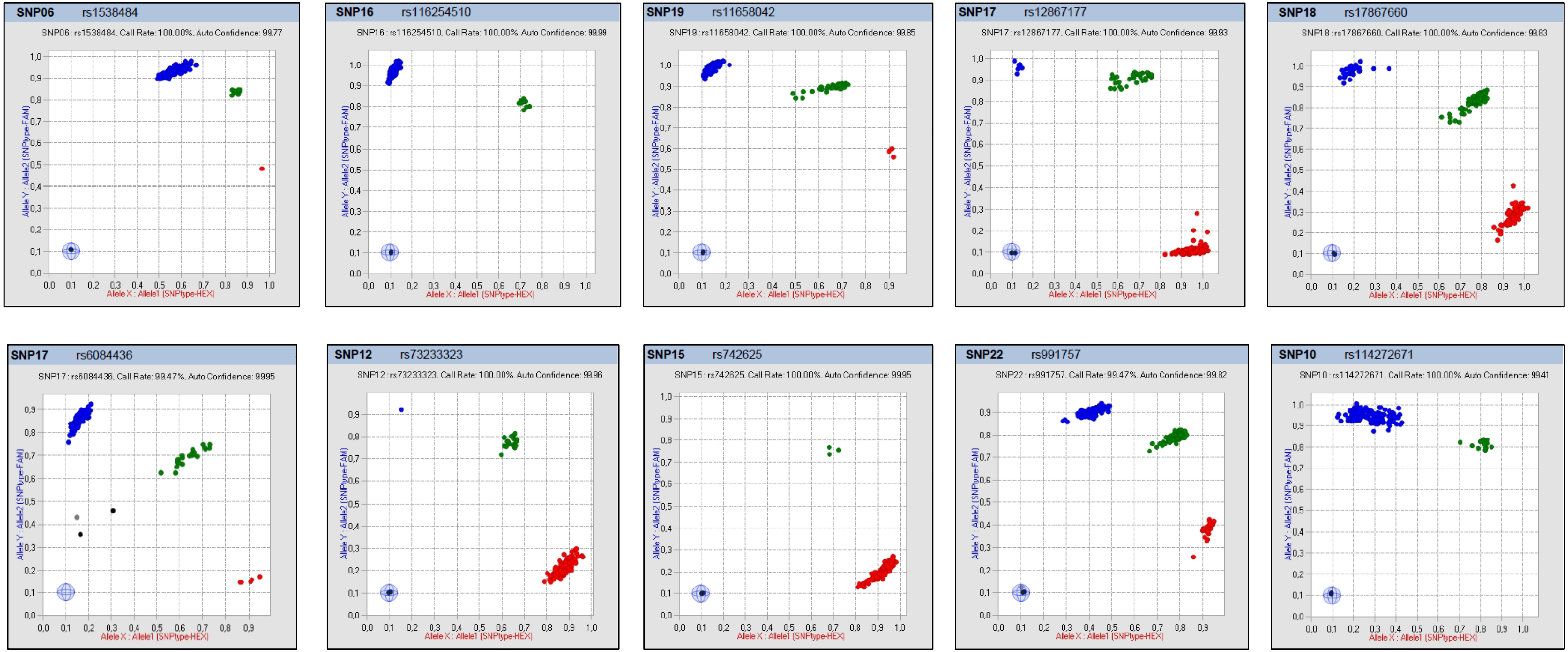

Supplementary Figure 2B

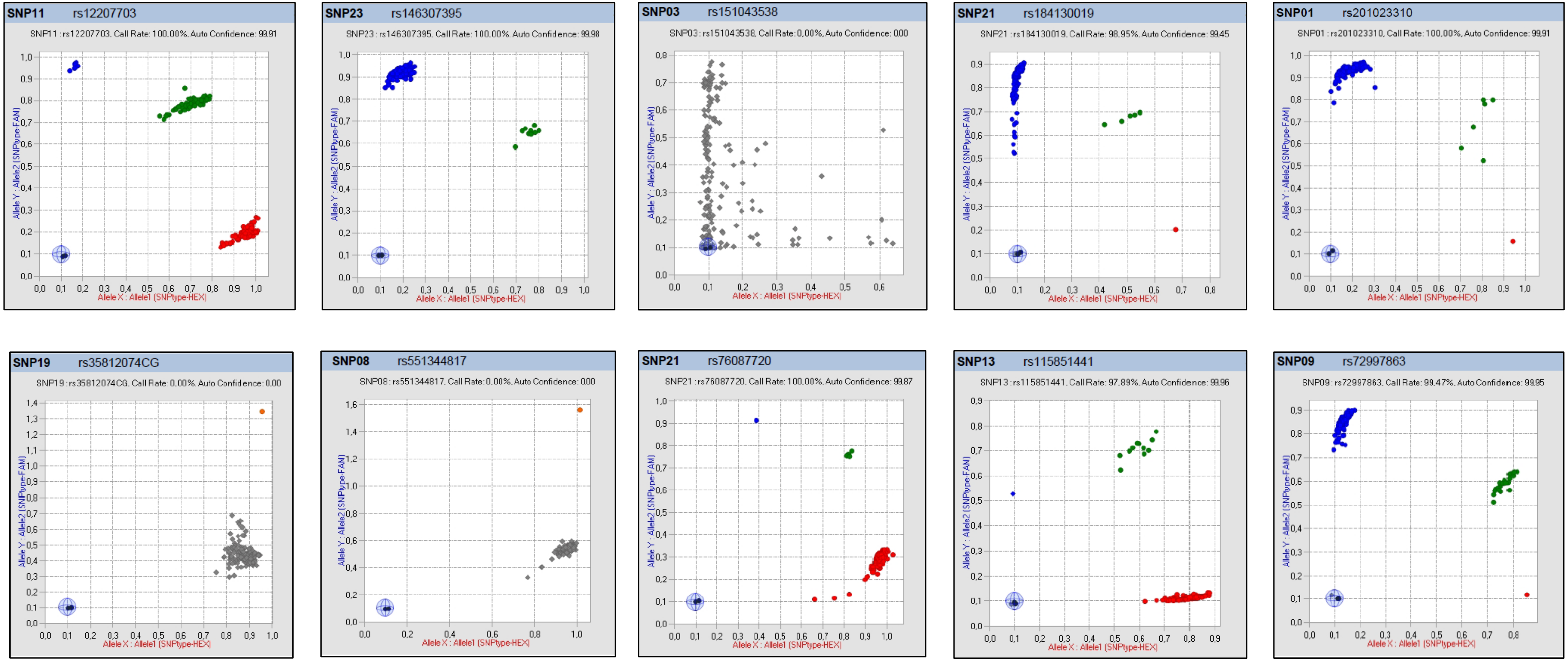

Supplementary Figure 2C

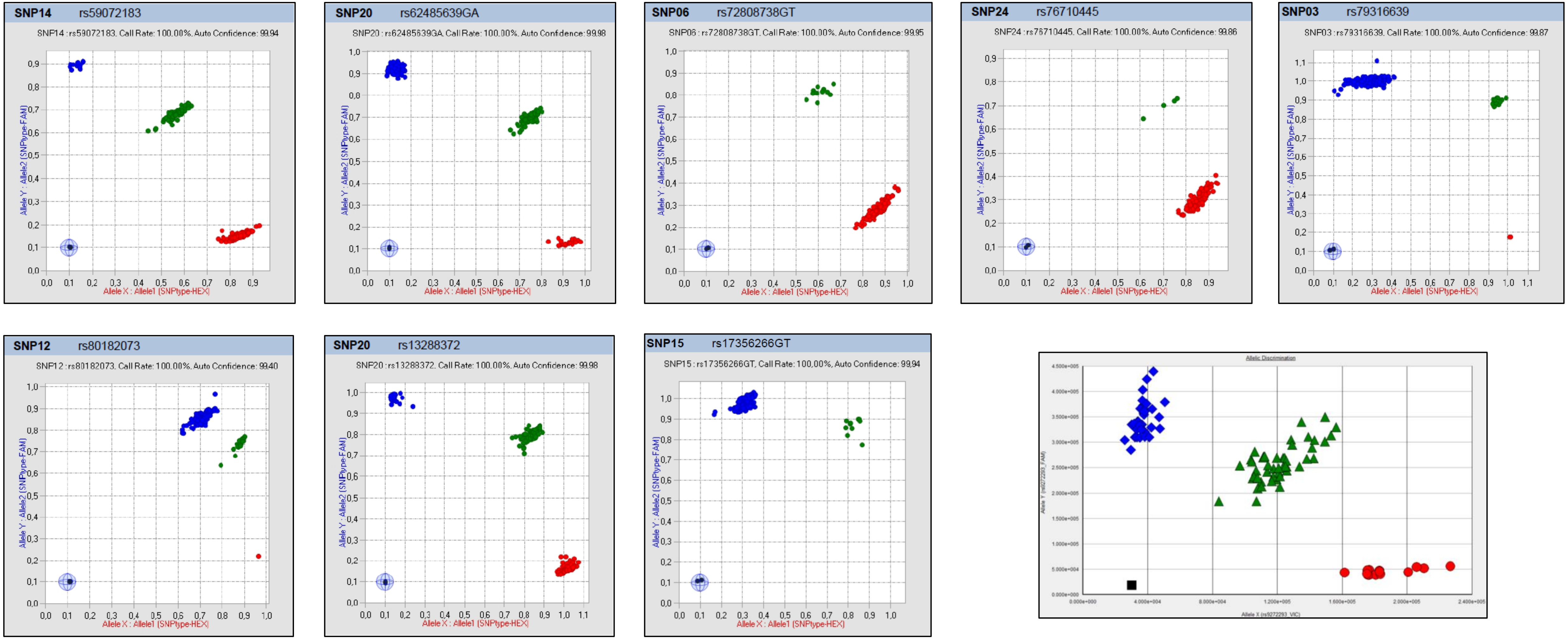

Supplementary Figure 3

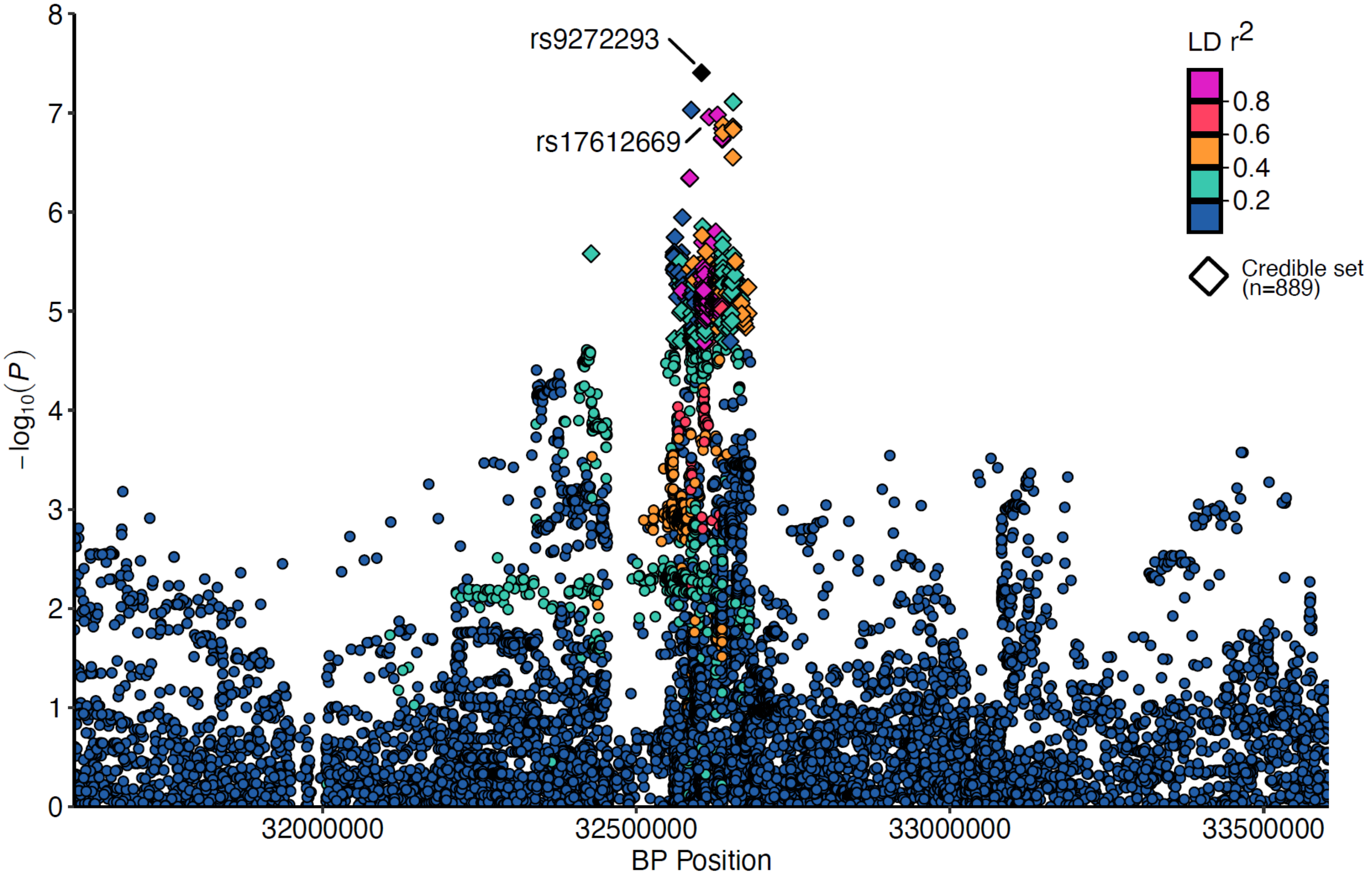

Supplementary Figure 4

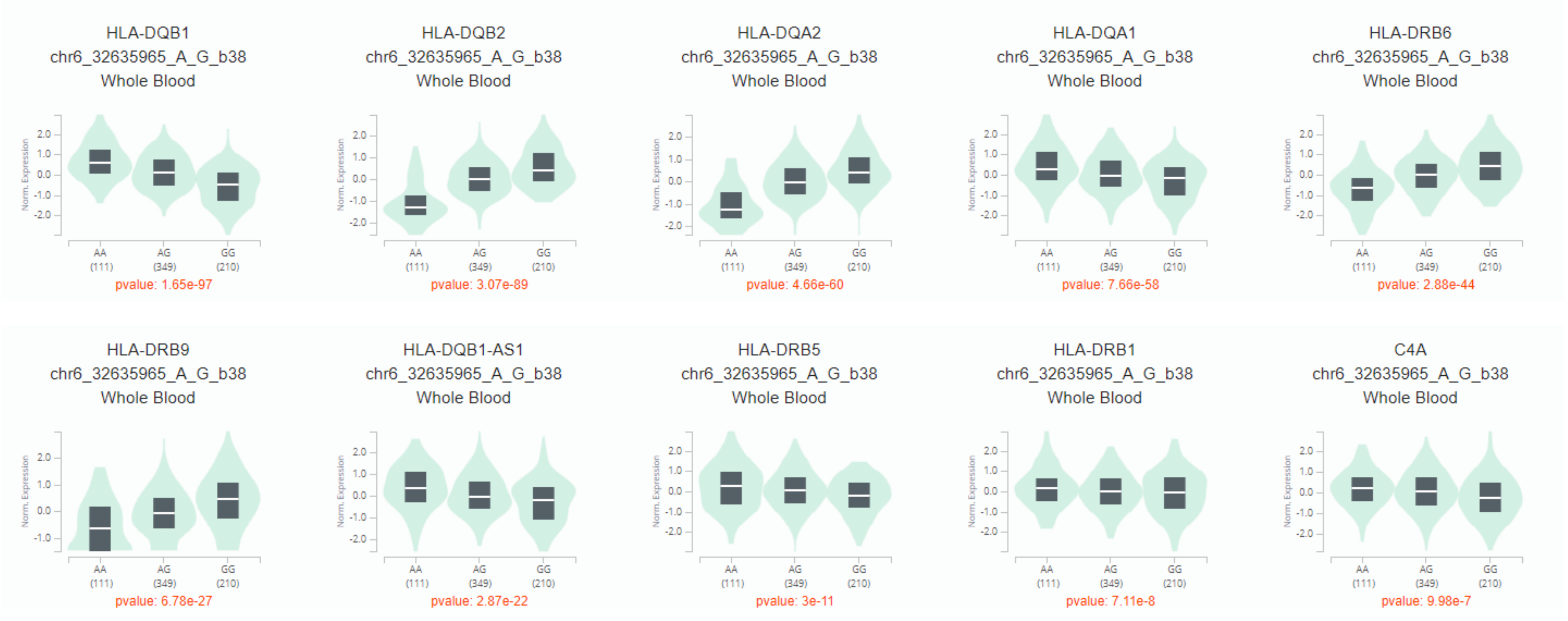

Supplementary Figure 5

A

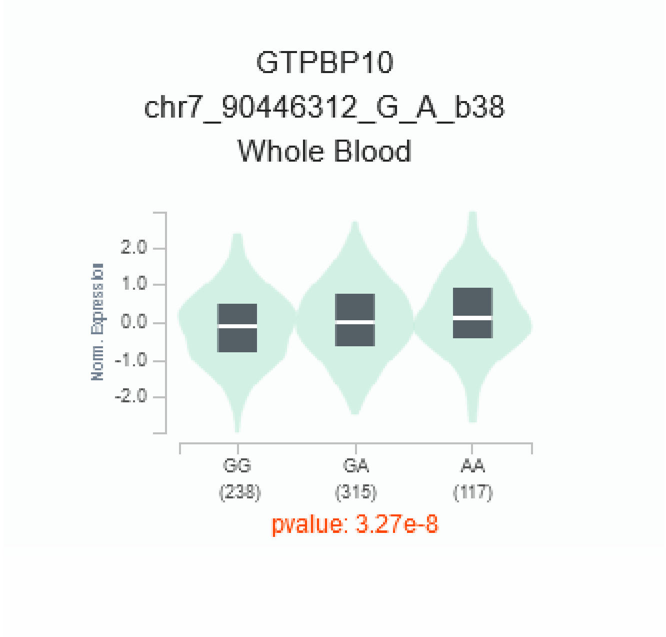

B

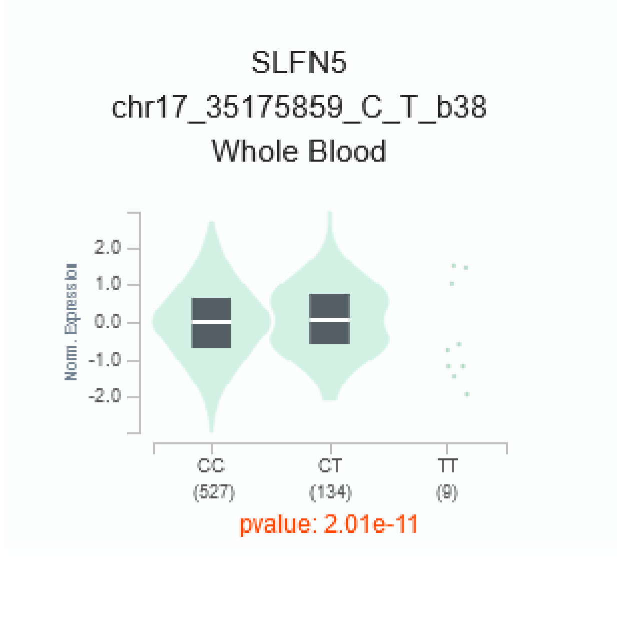

Supplement: Supplementary file 1 — Supporting information. [file JMV-97-e70195-s011.pdf]
