# Supplementary material for: Genome‐Wide Association Analyses of HPV16 and HPV18 Seropositivity Identify Susceptibility Loci for Cervical Cancer
Source: J Med Virol. 2025 Jan 31;97(2):e70195. doi: 10.1002/jmv.70195 (PMC11786146; doi:10.1002/jmv.70195)
Supplement: Supplementary file 16 — Supporting information. [file JMV-97-e70195-s005.docx]

**Supplementary Figures**

**Supplementary Figure 1: Study workflow.** Three genome wide association analysis (GWASs) were performed for HPV 16, HPV18 and HPV16/18 seropositivity. Numbers of cases and controls are shown. Six variants at four loci reached genome wide significance. A threshold of p < 5x10E-6 was set, leaving 170, 193, 677 suggestive variants from the HPV16, HPV18 and HPV16/18 seropositivity GWAS. Out of that, 29 independent variants (at R^2^ < 0.3) with a minor allele frequency of at least 1% (MAF ≥ 0.01) were selected for wet-lab validation. Four of them were excluded from further analysis due to bad clustering or failing the Hardy Weinberg equilibrium. The remaining 25 variants were taken for statistical analysis. 12 variants associated with cancer in our cohort in at least one subgroup (at p < 0.05). Bioinformatic analyses were performed for these variants.

**Supplementary Figure 2: Cluster plots after genotyping per variant.** Fluorescence intensities are shown with samples homozygous for Allele1 are in blue, heterozygous samples are in green and samples homozygous for Allele2 are in red. Negative template controls are black.

**Supplementary Figure 3: Fine-mapping of the locus +/-1 Mbp of rs9272293 using SuSiE.** -log10 p values are shown on the y-axis, with position on the x-axis. LD to the top SNP is shown by colors indicated in the key. Variants belonging to the credible set are shown in a diamond shape while all other variants are shown as circles.

**Supplementary Figure 4: eQTL analysis in whole blood for rs9272293 from GTEx.** Normalized expression values are shown on the y-axis, together with genotypes on the x-axis: rare homozygous AA, heterozygous AG and common homozygous GG. Sample numbers per group are written below the respective bars in brackets.

**Supplementary Figure 5:** **eQTL analysis in whole blood for (a) rs17867660 and (b) rs11658042 from GTEx.** Normalized expression values are shown on the y-axis, together with genotypes on the x-axis: (a) common homozygous GG, heterozygous GA and rare homozygous AA; (b) common homozygous CC, heterozygous CT and rare homozygous TT. Sample numbers per group are written below the respective bars in brackets.

**Supplementary Tables**

**Supplementary Table 1: Summary Statistics from HPV seropositivity GWASs.** Variants at p < 5x10E-6 are shown after GWAS analysis of a) seropositivity for HPV16 versus no HPV detected, b) seropositivity for HPV18 vs HPV18 negative, c) seropositivity for HPV16 and/ or 18 versus no HPV detected.

**Supplementary Table 2: Assay designs for the genotyped variants.** SNP name, name of the GWAS study that the variant was chosen from, genomic locus, position (GrCh37), common allele and rare allele, minor allele frequency in Europeans (MAF 1000G EUR), Fluidigm assay ID, Allele1 and Allele2 (from the Fluidigm design), orientation of the Fluidigm design (FWD = forward, REV = reverse), SNP_SEQ_STRAND – strand on which design is based, reference allele (from the Fluidigm design), sequences of allele specific primer for Allele1 (ASP1_SEQ) and Allele2 (ASP2_SEQ), sequence of locus specific primer (LSP_SEQ), sequence of primer for specific target amplification (STA_SEQ).

**Supplementary Table 3: Validation genotyping outcomes for top 25 variants.** SNP name, name of the GWAS study that the variant was chosen from, genomic locus, position (GrCh37), clustering information, Hardy-Weinberg equilibrium (HWE fail) testing (above 3.84 chi-square values considered failed), call rates and concordance rates (in %).

**Supplementary Table 4: Bioinformatic annotations for 12 validated variants.** a) SNP name, reference and alternate allele. eQTLs in whole blood from GTEx and eQTLGEN consortium. Chromatin status in HeLa cells, transcription factor binding motifs and ChIP-Seq experimental data and in HeLa cells from the Regulome Database. Reference and alternate allele sequence (+/-10 bp of the SNP), motifs specific for the reference allele and the alternate allele from TOMTOM MEME Suite^26^. Submitted sequences (+/-25 bp of the SNP) and transcription factor binding motifs changed between the alleles via the PERFECTOS-APE webtool. Changed motifs, nearest genes and functional annotations from Haploreg v4.2. Up to 10 genes in close vicinity (+/-1 Mbp) from UCSC genome browser at shown. b) Detailed results of motif changes reported in HaploReg are shown, with scores per allele, predicted TF-binding site and reference sequences.

**Supplementary Table 5:** Detailed results from eQTL search for the 12 variants of interest in eQTLGEN consortium. Reported are z scores and FDR corrected p-values.

**Supplementary Table 6: Genes taken for transcript analysis.** Fluidigm assay ID for the 36 candidate genes and housekeepers.

**Supplementary Table 7: rs9272293 predicts detection of gene transcripts.** Pearson correlation R values and two tailed p values are shown after testing for correlation between rs9272293 genotypes and detection of gene transcripts, either in all cervical tissue samples or when stratified by HPV status.

**Supplementary Table 8:** (A) Results from MAGMA gene set analysis with a Bonferroni corrected p-value in either of the three GWAS data sets. (B) Results of gene set enrichment analysis from GENE2FUNC in FUMA, Immunologic signatures (MsigDB c7), in the HPV16, HPV18, and HPV16/18 seropositivity analysis. (C) All the gene set enrichment results from GENE2FUNC for each of the three GWASs.

**Supplementary Table 9: Results from fine-mapping of the GWS locus.** Results after fine-mapping +/-1 Mb of the lead SNP rs9272293 via (A) SuSiE and (B) Rsparsepro. CSID and CS indicates the credible set number. PIP indicates posterior inclusion probability for each variant.

**Supplementary Table 10: Further results after logistic regression analyses.** Cervical intraepithelial neoplasia was separated into LSIL/low-grade (CIN1 + CIN2<30years) and HSIL/high-grade (CIN2≥30years + CIN3) subgroups. Invasive cervical cancer was further divided into squamous epithelial cell carcinoma and adenocarcinoma. High-risk dysplasia (CIN2≥30years + CIN3) and invasive cancer were also combined in joint analysis. HSIL & invasive cancers were further stratified by HPV status (HPV 16, HPV18, HPV16/18 and other hrHPV). CI, 95% confidence interval; OR, odds ratio for minor allele; p, p value from logistic regression analysis.

**Supplementary Table 11:** Detailed HaploReg v4.2 results per variant and linked variants at R^2^ ≥ 0.8.

**Supplementary Table 12:** Detailed RegulomeDB results per variant and linked variants at R^2^ ≥ 0.8.

**Supplementary Table 13:** Detailed PERFECTOS-APE results per top variant.

**Supplementary Table 14:** Detailed FORGEdb results per top variant.
